# ICA Extracts Epileptic Sources from fMRI in EEG-Negative Patients: A Retrospective Validation Study

**DOI:** 10.1371/journal.pone.0078796

**Published:** 2013-11-12

**Authors:** Borbála Hunyadi, Simon Tousseyn, Bogdan Mijović, Patrick Dupont, Sabine Van Huffel, Wim Van Paesschen, Maarten De Vos

**Affiliations:** 1 STADIUS Center for Dynamical Systems, Signal Processing and Data Analytics, Department of Electrical Engineering (ESAT), KU Leuven, Leuven, Belgium; 2 iMinds Future Health Department, Leuven, Belgium; 3 Laboratory for Epilepsy Research, KU Leuven, Leuven, Belgium; 4 Medical Imaging Research Centre, KU Leuven, Leuven, Belgium; 5 Laboratory for Cognitive Neurology, KU Leuven, Leuven, Belgium; 6 Department of Neurology, UZ Leuven, Leuven, Belgium; 7 Methods in Neurocognitive Psychology Lab, Department of Psychology, Cluster of Excellence ‘Hearing4all’, European Medical School, Carl von Ossietzky University, Oldenburg, Germany; 8 Research Center Neurosensory Science, Carl von Ossietzky University, Oldenburg, Germany; Universiteit Gent, Belgium

## Abstract

Simultaneous EEG-fMRI has proven to be useful in localizing interictal epileptic activity. However, the applicability of traditional GLM-based analysis is limited as interictal spikes are often not seen on the EEG inside the scanner. Therefore, we aim at extracting epileptic activity purely from the fMRI time series using independent component analysis (ICA). To our knowledge, we show for the first time that ICA can find sources related to epileptic activity in patients where no interictal spikes were recorded in the EEG. The epileptic components were identified retrospectively based on the known localization of the ictal onset zone (IOZ). We demonstrate that the selected components truly correspond to epileptic activity, as sources extracted from patients resemble significantly better the IOZ than sources found in healthy controls. Furthermore, we show that the epileptic components in patients with and without spikes recorded inside the scanner resemble the IOZ in the same degree. We conclude that ICA of fMRI has the potential to extend the applicability of EEG-fMRI for presurgical evaluation in epilepsy.

## Introduction

Functional magnetic resonance imaging (fMRI) combined with simultaneously recorded electroencephalogram (EEG) is a powerful multimodal approach, which potentially provides information with high spatial and temporal resolution. It has been extensively used for characterizing cognitive processes, e.g. [1], [2]. It is capable of localizing ictal, e.g. [3] and interictal epileptic activity, e.g. [4], [5] and can be applied as part of presurgical evaluation [6]. Standard analysis uses the timing of interictal discharges based on EEG to find regions with correlated blood oxygen level dependent (BOLD) signal changes recorded by fMRI within the standard general linear model (GLM) approach. However, marking interictal discharges on the EEG is time-consuming; furthermore, EEG does not always provide reliable information on the epileptic events. Neural activity of deep structures is not visible on EEG [7]–[9], moreover, the severe gradient and ballistocardiogram (BCG) artifacts due to the magnetic field in the scanner often make the interpretation of the EEG ambiguous [10], [11]. Several approaches exist to reduce such artifacts (for a comparison study, see [12]), however, residual artifacts still hinder interpretation. Most importantly, some patients might not show interictal activity during the limited time of the fMRI sessions. In a recent study only 37% of consecutively scanned patients showed clinically concordant epileptic spikes [13], thus, in the vast majority of cases, GLM-based analysis could not be carried out. It has been reported previously, that EEG-fMRI studies fail in 40–70% of cases [14], due to the reasons mentioned above or due to the lack of significant BOLD changes correlated to interictal activity. Therefore, there is a strong demand for techniques capable of localizing the epileptic activity without any information on its timing.

ICA was first proposed to process fMRI data by [15]. It has been widely used to explore resting state networks (RSNs), e.g. [16], task-related activations, e.g. [17], or to characterize the variability of the hemodynamic response [18]. In [19] a method was developed which automatically classifies the independent components (ICs) extracted from the fMRI time series as BOLD related, artifactual or noise related using IC-fingerprints constructed by 11 features.

ICA has also been applied to the fMRI time series recorded from epilepsy patients during ictal [3], [20], [21] and interictal [20 22, 23] period. The ICs were automatically grouped [3], [23] using the technique presented by [19]. Within the BOLD related class the epileptic ICs were identified based on spatial accordance with the seizure onset zone defined on intracranial EEG recordings or with the GLM EEG-fMRI activation maps and temporal correlation with the EEG-derived temporal regressor. Successful identification of the epileptic activity showed the potential of ICA in analyzing fMRI time series of epilepsy patients.

In summary, several studies have demonstrated that ICA can reveal BOLD sources related to epileptic activity. In all these works the selection of the component of interest relied on the known timing of the epileptic events. However, the application of ICA is of particular interest in those cases where no epileptic events can be identified during the recordings, consequently, an alternative to the traditional GLM based approach is needed. Therefore, the present study primarily focuses on focal epilepsy patients in whom no ictal or interictal activity could be identified on the EEG. Our aim is to demonstrate that ICA can estimate a component related to the epileptic network in such cases as well. We retrospectively identify the epileptic component based on its spatial correspondence to the known localization of the IOZ. To validate our findings, we analyze the data of a group of control subjects to investigate whether component maps resembling the IOZ exist in healthy individuals.

## Materials and Methods

### 2.1 Data Acquisition and Preprocessing

The study was approved by an independent ethical standards committee on human experimentation of the University Hospitals Leuven and written informed consent was obtained from all participants.

A total of 28 patients were included in this study based on the following criteria: (1) consecutive adults who underwent a full presurgical evaluation for refractory focal epilepsy between August 2010 and January 2012, including seizure history, neurological and physical examination, interictal and ictal scalp EEG-recordings, video-analysis of seizures, high-resolution MRI of the brain, interictal and ictal single-photon emission computed tomography (SPECT) and subtraction ictal SPECT co-registered to MRI (SISCOM), neuropsychological assessment, and when available interictal 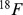-fluorodeoxyglucose Positron Emission Tomography (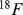-FDG PET) and intracranial EEG-recordings; and (2) concordant data pointing to one epileptic focus using all presurgical investigations. For clinical details of the patients, see Table 1. The ictal onset zone was defined as follows. In 11 patients who underwent epilepsy surgery with successful outcome (ILAE classification 1–4), we considered the region of ictal hyperperfusion, determined by SISCOM, inside the resection zone as the IOZ. The hyperperfusion on SISCOM was thresholded with 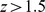. This threshold was shown to be optimal for localizing the epileptogenic zone [24]. In patients, planned or ineligible for surgery because the epileptogenic zone was within eloquent cortex, the IOZ was determined as the SISCOM hyperperfusion cluster (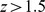) in a manually outlined hypothetical resection area, based on multidisciplinary clinical consensus using all noninvasive and invasive data except EEG-fMRI results. Note that an injection delay of less than 20 s was shown to correlate with correct localization [25], whereas the median injection delay was 16.5 s in our dataset. In cases where injection delay and propagation did occur, visual recognition of so-called “hourglass” patterns helped differentiate between onset and propagation areas [26]. Nevertheless, by confining the IOZ to the hyperperfusion region inside the actual or hypothetical resection zone, we avoided the inclusion of hyperperfusion clusters corresponding to areas of propagated ictal activity. Multimodal concordant seizure focus localizing data increase the likelihood of benefit from surgical treatment [27]–[29]. Since patients were selected for concordant localizing data, we ensured not to rely on a single testing modality.

**Table 1:** Clinical description of the patients.

Patient	Gender/age/age at onset	Ictal onset zone	Etiology	SPECT injection time/seizure duration (s)	Structural lesion	icEEG concordant	Resection	Outcome ILAE 2001 (1–6)	Follow-up time after surgery (m)	Pathology	Spikerate (spikes/h) during fMRI
1	F/38/12	R temporal	CNS infection	15/76	Ischaemic R parietotemporooccipital lesion	N/A	Planned				1171
2	M/24/2	L posterior temporal	Postsurgical gliosis	42/47	L temporal gliosis after L amygdalohippocampectomy	N/A	Planned				0
3	M/26/22	L anterior temporal	FCD	12/42	Normal	N/A	Yes	1	25	FCD	0
4	M/34/18	R anterior temporal	HS	10/58	Normal	N/A	Yes	1	14	HS	0
5	F/23/9	L anterior temporal	DNET	15/54	L temporal DNET	N/A	Yes	2	18	DNET	187
6	M/24/18	R anterior temporal	Unknown	53/90	Normal	N/A	Refusal				0
7	F/56/8	R parieto-occipito-temporal	Sturge-Weber	16/33	Superficial angioma and hemiatrophy R posterior convexity	N/A	Planned				156
8	M/20/7	L parieto-temporal	Unknown	94/139	L parietal gliosis after surgery	Yes	Overlap eloquent cx				15
9	F/34/15	R frontal	FCD	3/20	Subtle R frontal FCD	Yes	Yes	2	13	Gliosis, micraglia activation, neuronal loss	853
10	F/55/38	L anterior temporal	HS	38/73	L HS	N/A	Yes	1	18	HS	156
11	M/39/31	L temporal	DNET	27/64	L temporal DNET	N/A	Yes	1	13	DNET	0
12	M/17/3	R anterior temporal	CNS infection+HS	5/76	R HS	N/A	Yes	1	15	HS	0
13	M/44/12	R temporal	HS	21/72	R HS	N/A	Yes	1	10	HS	0
14	M/27/22	L anterior temporal	Unknown	10/94	Normal	Yes	Planned				36
15	F/21/12	L temporal	Post-traumatic	23/45	L HS and biparietal contusions	N/A	Planned				0
16	M/26/1	L mesial frontal	Tuberous sclerosis	3/29	L frontal gliosis and FCD Lfrontal, biparietal andbioccipital	N/A	Planned				710
17	M/40/27	R mesial temporal	CNS infection+FS	21/60	R HS	N/A	Refusal				0
18	F/61/12	L frontal	FCD	3/18	L frontal FCD	N/A	Overlap eloquent cx				915
19	F/29/18	R occipital	Unknown	26/122	Normal	N/A	Overlap eloquent cx				0
20	M/19/10	R parietal	Leptomeningitis	14/68	Normal	Yes	Yes	1	13	Lepto- meningitis	0
21	F/31/9	L perirolandic	Stroke	18/80	Ischaemic L frontoparietallesion, L temporal hypoplasia	N/A	Overlap eloquent cx				0
22	F/63/21	R mesial temporal	FS+HS	30/162	R HS	N/A	Yes	1	14	HS	0
23	F/39/27	R ventrotemporal	Cavernous angioma	10/52	R temporal cavernous angioma	N/A	Yes	2	11	Angioma	0
24	M/45/36	L parieto-temporal	Unknown	17/45	Normal	N/A	Overlap eloquent cx				0
25	M/29/2	R frontal	Postsurgical gliosis	4/25	R frontal gliosis	Yes	Planned				0
26	M/30/15	R temporal	Unknown	12/58	Normal	N/A	Planned				733
27	F/23/2	L temporal	FCD	43/158	L temporal FCD	N/A	Overlap eloquent cx				0
28	F/33/7	L anterior temporal	FS+HS	61/92	L HS	N/A	Planned				1

BOLD fMRI data were acquired in the selected patients using a 3T MR scanner (Intera or Achieva, Philips) with a whole brain single-shot T2* gradient-echo Echo Planar Imaging sequence (TE = 33 ms, TR = 2.2–2.5 s, voxel size 2.6×3×2.6 mm). The images were realigned, slice-time corrected, normalized to MNI space and spatially smoothed with an isotropic Gaussian kernel of 6 mm full width at half maximum using SPM8 software (Wellcome Department of Cognitive Neurology, London, UK). Scalp EEG was simultaneously recorded with a 64 or 32 channel MR-compatible EEG cap (Brain Products, Munich, Germany) or a 24 channel electrode set (Ives EEG solutions inc). The EEG signals were amplified (BrainAmp amplifier, sampling rate 5000 Hz, resolution 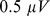) and transmitted outside the scanning room. From each patient 2 to 4 sessions were recorded, which lasted on average 12 minutes, ranging from 10 to 22 minutes each. The patients were asked to rest with closed eyes. The EEG was band-pass filtered offline between 1 and 50 Hz, using windowed sinc FIR filters with a Hann window, gradient artifacts were removed using a realignment-parameter informed template subtraction algorithm ([30], Bergen plug-in for EEGLAB, Bergen fMRI Group, Bergen, Norway), and BCG artifacts were subtracted using a dynamic average artifact template subtraction method ([31], Brain Vision Analyzer software, Brain Products, Munich, Germany). After artifact correction, interictal epileptic spikes, if present, were visually marked by a neurologist based on the EEG. Interictal epileptic activity was identified in 13 out of 28 patients. Clinically concordant interictal spikes were marked in 11 out of 28 patients during the EEG-fMRI recordings. Additionally, in 3 cases, contralateral or bilateral spikes (patient 14, 19 and 26) and in 2 cases pathological slow wave activity were identified (patient 10 and 24).

Functional images were also acquired from 12 healthy controls (4 male, 8 female, age range 21–56, mean age 34.4) using the same protocol and preprocessing as described above.

Scans recorded during large movements of the patients and controls were discarded: the largest consecutive series of scans were retained where the momentary displacement did not exceed 1 mm. Additionally, movement related effects were estimated by constructing nuissance regressors based on the realignment parameters, were fitted to the fMRI time series using SPM8, and were finally regressed out from the fMRI time series. A combined gray and white matter mask obtained in SPM8 was applied to remove voxels within cerebral spinal fluid structures and resected lesions.

### 2.2 Independent Component Analysis of fMRI Time Series

The measured fMRI time series is the result of the mixture of ongoing neural activity, artifacts and noise. Assuming that the activity of interest has a fixed spatial pattern which is independent of the ones related to other underlying processes, ICA separates them in the following way:
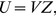
(1)where 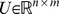 is formed by the component voxel values where 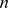 is the number of independent spatial components, which equals the length of the time series, and 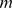 is the number of voxels. 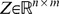 contains the measured fMRI time course of all voxels and 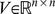 is the unmixing matrix. The columns of the mixing matrix 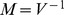 are the time courses associated with each IC. In such formulation, the ICA of the fMRI will deliver as many independent components as the number of samples in the time series. However, the data usually can be explained by a smaller number of underlying processes. In order to reduce the number of components, the temporal dimension of each time series is reduced by principal component analysis to an estimated optimum. The optimal number of ICs was estimated automatically for each session of each patient using the minimum description length (MDL) criteria ([32], as implemented in the GIFT toolbox (http://www.nitrc.org/projects/gift/). For each patient and control, the maximum of the optimal number of ICs over all sessions were taken, resulting in 53.3±13.1 components across the individuals. Individual sessions were first reduced in dimension, then concatenated into an aggregate dataset and reduced again to obtain the optimal dimension. Subsequently, independent component analysis was performed on each aggregate dataset using the infomax algorithm [33]. Spatial maps and time courses of individual sessions were backreconstructed using the aggregate mixing matrix [34]. Finally, the mean spatial map over all sessions were considered for each patient and control.

### 2.3 Identification of the Epileptic IC

In order to identify ICs corresponding to the epileptic network, the procedure below was followed. The component voxel values were converted to z-scores by extracting the mean and dividing by the standard deviation of all voxel values within the component map. A threshold of 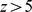 was used to create component activation maps. Finally, the component activation maps were ordered based on their overlap with the IOZ
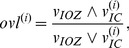
(2)where 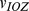 and 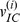 denote the set of voxels within the IOZ and the set of suprathreshold voxels of the 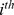 IC, respectively. Note that suprathreshold voxels may occur at several distant regions in the brain, i.e. various distant groups of adjacent suprathreshold voxels will form various activation clusters throughout the brain.

The high threshold of 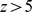 was chosen in order to achieve high specificity and omit ICs which show negligible overlap with the IOZ. Note that the overlap measure defined here is symmetric, unlike others used in the literature (e.g. considering an overlap larger than 10% of the extent of GLM-activation map a match [23]). As such, our measure is not sensitive to the chosen threshold for the IC maps: lowering the threshold increases the extent of the overlap but increases the set of activated voxels outside the IOZ as well. We will consider an overlap large if its value exceeds 5%. As the average extent of the IOZ over all patients was approximately 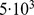 voxels, the average number of suprathreshold voxels in the ICs was 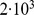 and the total number of voxels after masking out CSF structures exceeds 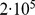, an overlap larger than 5% is unlikely to occur by chance.

ICs showing a significant temporal correlation (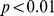, corrected for multiple comparisons using Bonferroni’s method) with the realignment parameters and showing spatial patterns such as outstreched activation clusters along the surface of the brain or several scattered clusters, assessed by visual inspection, were considered as head movement related artifacts and were excluded. The remaining sources, which overlap with the IOZ, are candidate ICs, i.e. potentially related to epileptic activity. Finally, the candidate IC showing the largest overlap with the IOZ will be called the epileptic IC (eIC).

### 2.4 Validation with Control Subjects

In order to assess the risk of finding false overlaps (coincidental overlap with an IC which is not related to epileptic activity), the overlaps between the ICs of each control and the IOZ of each patient were analysed. For each IOZ, the IC showing the largest overlap with it was selected from each control. The values of these overlaps (28 times 12 values in total) were then averaged to obtain an expected false overlap extent for each of the 28 IOZ. The expected false overlap extents were then compared to the overlap between the IOZ and the eIC in the corresponding patient.

### 2.5 Analysis of the eIC Time Course

For the 13 patients who had interictal activity during the fMRI recordings, the timing of the epileptic events were compared to the time courses of all ICs. Reference BOLD time courses were created by convolving the timing of epileptic events with the canonical hemodynamic response function.

## Results

### 3.1 Description of the Epileptic ICs

**Figure 1:**
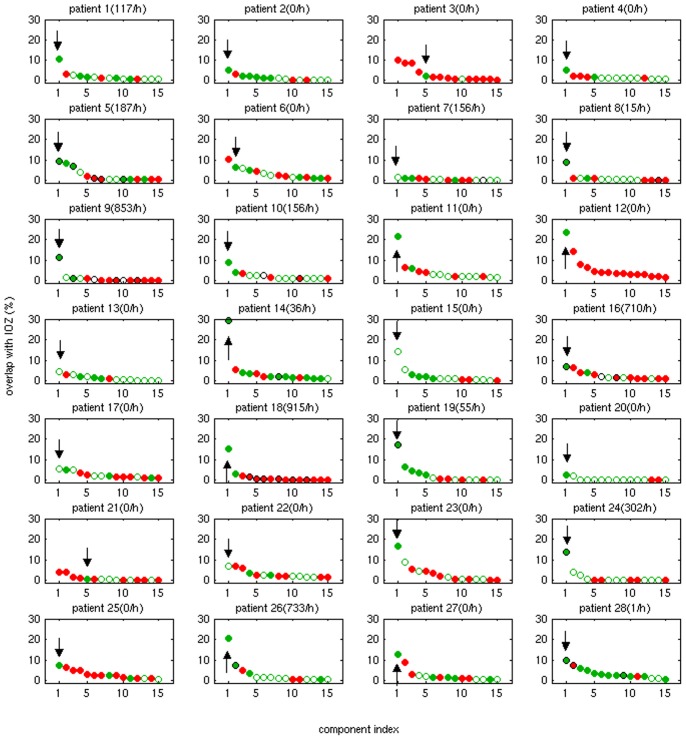
The extent of overlap of ICs with the IOZ for each patient. The number of spikes marked on the EEG inside the scanner is indicated in brackets next to the patient number. For each patient 15 ICs with the highest overlap are plotted in descending order. The eICs are indicated with an arrow. If the voxel with maximal z-score belongs to the cluster which overlaps with the IOZ, the IC is marked with a filled green circle. If the voxel with maximal z-score belongs to another cluster, the IC is marked with an empty circle. ICs resembling movement artifacts are marked in red. ICs which are significantly correlated to the timing of the epileptic activity are marked with an outer black circle additionally.

**Figure 2:**
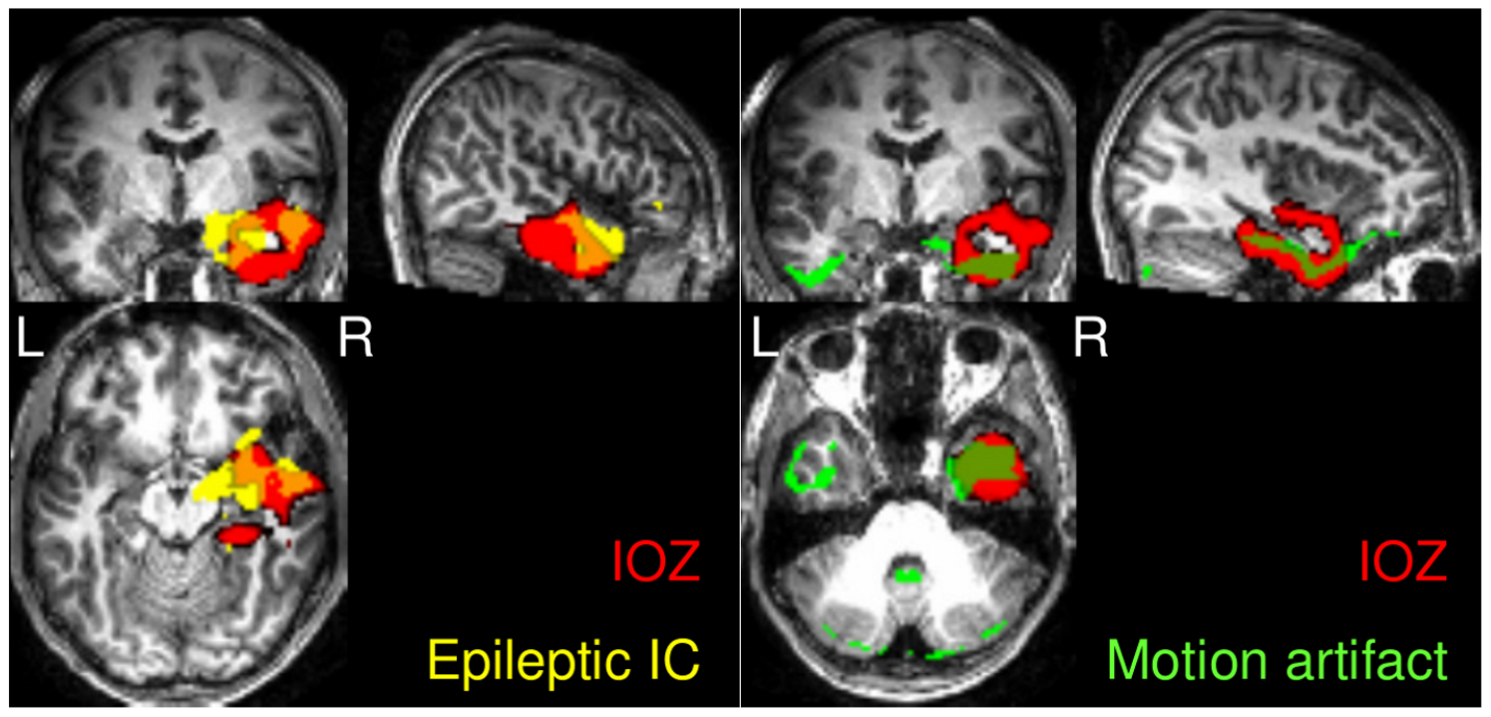
In several patients multiple ICs overlapped with the IOZ. Here the example of patient 12 is shown. The IC marked with green is a head movement related artifact, its time course showing significant correlation to the realignment parameters. Therefore, only the IC marked in yellow is considered to be a candidate eIC. Note the extensive overlap (23%, marked in orange) with the IOZ (marked in red) and that in this patient no interictal spikes where recorded in the EEG.

Figure 1 shows the extent of overlap between the ICs and the IOZ for each patient. If the voxel with maximal z-score belongs to the cluster which overlaps with the IOZ, the IC is marked with a filled green circle. If the voxel with maximal z-score belongs to another cluster, the IC is marked with an empty circle. ICs which are significantly correlated to the timing of the epileptic activity are marked with an outer black circle additionally (see section 3.3). ICs corresponding to movement artifacts are marked in red. Figure 2 shows the example of patient 12, a case without interictal spikes recorded in the EEG, where several different ICs showed large overlap with the IOZ. However, only one of them is considered to be a candidate IC, as the others are due to movement related effects. The eIC of each patient, selected as the IC showing the largest overlap with the IOZ from the candidate ICs, is marked with an arrow on Figure 1.

**Figure 3:**
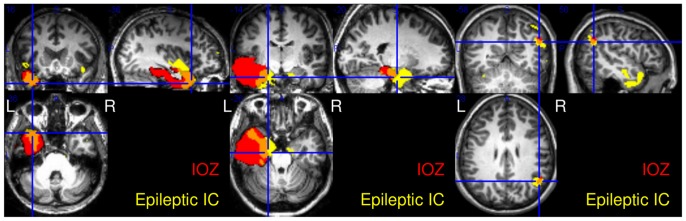
Examples of patients, in whom no interictal spikes were recorded in the EEG. Patient 11 and patient 27 (left and middle): in both cases the eIC (in yellow) shows large overlap (in orange) with the IOZ (in red), 22% and 13%, respectively. Patient 20 (right): Despite the quantitatively small overlap, the eIC is highly informative with respect to the IOZ. In all three cases the voxel with maximal z-score, indicated by the crosshair, is within the cluster overlapping with the IOZ.

The average overlap between the eIC and the IOZ is 10.6%±7.2, more particularly, 12.5%±7.0 and 9.60%±7.1 for patients with and without interictal activity, respectively. The difference between the patient groups is insignificant (p = 0.19, F-test). Figure 3 shows 2 examples, patient 11 and patient 27, where no interictal spikes were present in the EEG. In both cases the eIC shows an extensive overlap with the IOZ, and the voxel with maximal z-score is within the cluster overlapping with the IOZ.

The location of the voxel with the maximal z-score is particularly important from various aspects. First, a spatial map might consist of various activation clusters, however, the extent and the number of these clusters are threshold dependent, unlike the location of the voxel with the maximal z-score. Therefore, if the voxel with the maximal z-score belongs to the cluster overlapping with the IOZ, the assumption that the selected eIC is really epileptic gains further support. Furthermore, it shows that these eICs are informative with respect to the IOZ, i.e. if in a future application the eIC is selected automatically, the voxel with the maximal z-score indicates the IOZ.

The cluster within the eIC which overlaps with the IOZ contains the voxel with the largest z-score in 23 out of 28 patients. In patients 13 and 17 with temporal lobe epilepsy the eIC is a symmetrical bitemporal activation map, where the voxel with the maximal z-score is in the contralateral temporal lobe. The eICs of patients 7, 15 and 22 the eIC are contaminated with movement artifacts, which showed correlation with the realignment parameters below significance level. However, in all patients, even if it does not hold for the eIC, there exists a candidate IC, where the voxel with the largest z-score is within the cluster overlapping with the IOZ.

The eIC shows small (i.e. <5%) overlap with the IOZ in 5 patients (patients 3, 7, 13, 20 and 21). Figure 3 shows such an example, the eIC and its overlap with the IOZ of patient 20. Despite the quantitatively small overlap, the IC is highly informative with respect to the IOZ. Similarly, in patient 3 and 21, where the voxel with the maximal z-score is within the overlapping cluster, the eIC is indicative of the IOZ. Note that in patients 7 and 13 there is a small overlap between the eIC and the IOZ, moreover, the voxel with the maximal z-score is not indicative of the IOZ.

### 3.2 Validation with Control Subjects

The extent of the overlap between the IOZ and the eIC, 10.6%±7.2 on average, was significantly larger than the expected false overlap, 5.6%±2.4 on average (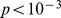, Wilcoxon signed rank test). However, control ICs showed a larger overlap than patient ICs in 7 out of 28 cases, in patients 3, 7, 13, 16, 17, 21 and 25. In patients 16 and 25 the IOZ is in the mesial frontal cortex and right frontal cortex close to the midline, respectively. In controls a map resembling the resting state network related to executive control [4] was consistently found, which showed large overlap with the IOZ of these patients. In the other 5 cases the eIC showed small (i.e. <5%) overlap with the IOZ or the voxel was not indicative to the IOZ. The question raises, whether the epileptic network was inactive in these patients, or ICA failed to decompose it correctly.

In order to make sure that the significant difference in overlap between patients and controls above was not due to some confounds, further tests were performed. First, the above procedure was repeated after excluding 6 patients who had surgical lesions, resulting in significantly larger overlap between patient ICs and IOZ (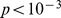). Second, the 12 controls were gender and age-matched to 12 patients and the overlaps of patient ICs were compared to the overlaps of the corresponding control ICs. Patient ICs overlapped significantly more with the IOZ (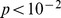). Finally, the severity of the patients’ and controls’ movements was compared based on the realignment parameters. There was no significant difference between the maximal displacement of patients and controls.

In summary, patient ICs showed a significantly larger overlap with the IOZ than control ICs did on the group level. On the individual level, in 75% of the cases an eIC was found which showed larger overlap with the IOZ than the extent of a coincidental overlap.

### 3.3 Analysis of the eIC Time Course

ICs showing significant correlation with the epileptic activity are marked with a black outer circle on Figure 1. The time course of the eIC was significantly correlated to the reference BOLD signal in 6 cases, and significantly anticorrelated in 2 cases. Interestingly, in most cases more than one IC showed significant correlation with the regressor. Several components exist which are significantly correlated both to the epileptic events and to head movement.

Although our definition implies that there is exactly one eIC per patient, there might exist multiple meaningful components. Both spatial overlap with the IOZ and temporal correlation to the interictal epileptic discharges were found in multiple ICs in several patients.

**Figure 4:**
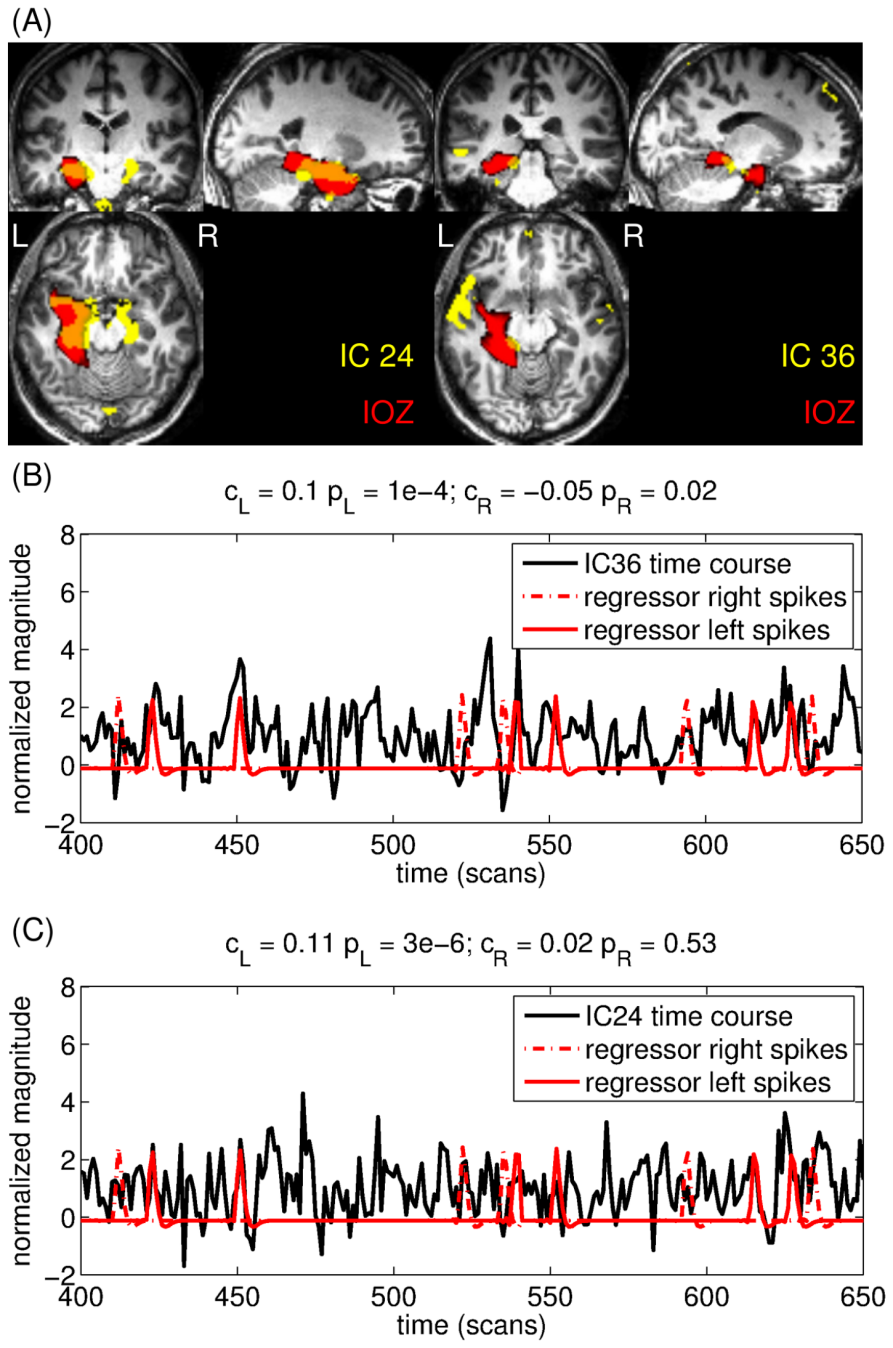
There are 2 ICs showing extensive overlap with the IOZ in patient 14. (A.) Left: IC # 36 has a left lateralized activation map and its time course (B.) is correlated with the reference BOLD signal based on the left-sided interictal spikes and anticorrelated to the regressor based on the right-sided spikes. (A.) Right: IC # 24 has a bilateral activation map and its time course (C.) is correlated with the reference BOLD signal based on the left-sided interictal spikes. For better visualization, only a short segment of the time courses are shown. The correlation coefficients and the significances are shown above the graphs.

In case of patient 14 one IC shows a lateralised, the other a bilateral activation (see Figure 4). On the EEG of this patient both left sided and right sided spikes were marked. IC # 36 has a left lateralized activation map and its time course is significantly correlated to the reference BOLD signal derived from the left sided spikes and anticorrelated to the one derived from the right sided spikes (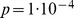 and 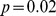, respectively ), while IC # 24 has a bilateral activation map and its time course is significantly correlated to reference BOLD signal derived from the left sided spikes (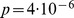). This suggests that the different ICs correspond to 2 different aspects of epileptic activity, which only partially overlap.

## Discussion

Several studies have shown the clinical usefulness of EEG-fMRI in localizing interictal epileptic activity in the brain. The main disadvantage of this technique is that it relies on the timing of epileptic activity as read on the EEG, which is, in a majority of the cases, not available or not reliable.

A few studies have already presented promising approaches in this direction applying a variety of model based and/or data-driven techniques. One group of methods aims at extracting meaningful information from the simultaneously recorded EEG without recurring to the identification of epileptic events per se. In [14] EEG voltage maps were created by averaging interictal epileptiform discharges recorded during long-term clinical monitoring outside the scanner. Subsequently, the correlation of this map with the EEG recordings inside the scanner was computed for each time frame. The time course of this correlation coefficient was used as a regressor for fMRI analysis to map hemodynamic changes related to these epilepsy-specific maps. The method revealed concordant activation maps in 78% of the cases with previously inconclusive EEG-fMRI results. Alternatively, [20] computed spectral metrics from independent components extracted from the EEG recorded inside the scanner. These spectral metrics express different transfer function models between EEG and BOLD signals. The resulting time courses are convolved with a canonical HRF and used as regressors of interest in a GLM analysis of the fMRI data. The advantage of this approach lies within providing insight into the link between neuronal and hemodynamic signals. On the other hand, another emerging group of approaches analyse solely fMRI data and do not require the simultaneous recording of EEG. Temporal clustering analysis (TCA, [36]) and its improved version, 2dTCA, [37] was developed to detect multiple activation patterns of irregular, transient behavior. Further, a new wavelet basis, called activelets, was developed based on the linear approximation of the balloon model of the HRF [38]. Activelets were applied in [39] to detect transient activations related to epileptic spikes on the fMRI voxel time series. Both methods showed promising results in simulation studies, however, only limited validation on EEG-negative patient data was performed.

We apply a data-driven methodology, namely ICA, which can estimate neural sources purely from the fMRI data without any information on the temporal pattern of their activation. Our aim was to demonstrate that a source related to the epileptic network exists among the components extracted by ICA. It has been shown previously by [23] that ICA can find epileptic sources, where the timecourse of the epileptic source is correlated to the timing of epileptic events, and the spatial map is similar to the GLM-based activation map. To our knowledge, it is shown here for the first time that the epileptic network can be found with ICA even in patients where no ictal or interictal activity was present on the EEG during the fMRI recordings. This result suggests that the epileptic network is continuously active in the brain even if it is invisible on the EEG.

The epileptic source was selected from all the estimated components based on spatial overlap with the a-priori known IOZ. The identification involved an arbitrary 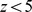 threshold on the spatial maps. Varying this threshold, however, had limited influence on our main findings. Components related to head motion artifacts were excluded prior to the selection of the candidate epileptic components. Several other types of artifacts were described in the literature and were recognized among our components. Due to the lack of available tools these components were not excluded automatically. Visual inspection nevertheless confirmed that the selected epileptic components were not originated from artifacts.

Additionally to the spatial overlap with the IOZ, we demonstrated in several other ways that the selected eIC is truly epileptic. The voxel with the maximal z-score belonged to the cluster overlapping with the IOZ in 23 out of 28 cases, i.e. the selected component is not a threshold dependent coincidental overlap. This is especially relevant information in those cases, where the overlap between the eIC and the IOZ was small (<5%). Considering this aspect, the criteria for selecting the eIC could be redefined in the following way: eIC is the candidate (not artifact related) IC showing the largest overlap with the IOZ where the overlap is at least 5% or the overlapping cluster contains the maximal z-score. These alternative criteria would select different eICs only in 2 patients, hence, this does not have an implication on our main findings. As further validation, we showed that eICs extracted from patient data overlap significantly better with the IOZ than ICs extracted from healthy individuals. Finally, in the majority of cases where interictal activity was reliably identified on the EEG, the eIC time course correlated with the timing of this activity. Although it has been reported in the literature that an epileptic IC with both spatial correspondence and significant correlation with epileptic activity was found in up to 90% of focal epilepsy patients, in these studies the patient group was preselected for concordant GLM-fMRI results. In the current study no such preselection was made. Simultaneous non-invasive and invasive recordings [7] have provided evidence that many spikes recorded with invasive techniques can not be detected with surface electrodes. Therefore, we argue that components showing high overlap with the IOZ might reflect a certain type of epileptic activity in EEG-negative and non-correlated cases as well, despite the fact this activity is invisible on the EEG.

As our aim was to demonstrate the existence of an IC informative of the IOZ, we selected, by definition, one eIC. However, note that in some patients multiple candidate ICs were found showing extensive overlap with the IOZ, which are potentially all meaningful. Similarly, it has been reported, that ICA finds multiple components corresponding to the same functional network [40], [41] in resting state fMRI. The question arises whether in such cases the same network is captured partially by various components, or the components reflect actually different underlying neurophysiological processes. In most cases multiple ICs correlated with the spike-derived regressor, including ICs which showed no or negligible overlap with the IOZ. This might mean that the onset and propagation zones are separated in different components. In case of one patient it was possible to match the different epileptic fMRI components to partially different types of electrophysiological interictal activity. A thorough analysis including all patients should be carried out to further investigate this aspect. Furthermore, in patients where the eIC showed small overlap with the IOZ, it is uncertian whether the epileptic network was inactive, or ICA failed to decompose it corretly. This ambiguity is an inherent limitation in data-driven methods.

Our ICA procedure is not an automatic identification method, as the epileptic source was chosen retrospectively, according to the known localization of the IOZ. For a prospective use of ICA to identify epileptic activity on the fMRI, an automatic technique for selecting the eIC is required, which does not use such a-priori information. [19] developed 11 features distinguishing BOLD related ICs from non-BOLD related, artifactual ICs. These features were used successfully by [23] to reduce the number of potential epileptic ICs. The epileptic IC best resembling the GLM-based activation maps was visually selected from this reduced set. Future work will be carried out to fully automate this procedure, by characterizing the eICs revealed by our approach and to develop new features distinguishing epilepsy related ICs from other BOLD related sources. Nevertheless, assuming that the epileptic network can be selected automatically, the voxel with the maximal z-score could identify the cluster overlapping with the IOZ. In our database the vast majority of patients had an eIC in which the voxel with the maximal z-score belonged to the cluster overlapping with the IOZ. Note that all patients had at least one candidate IC with such property, and these ICs are all potentially useful for identifying the IOZ. This implies that ICA of fMRI could facilitate the identification of the IOZ even in EEG-negative patients. Therefore, it has the potential to extend the applicability of fMRI assisting presurgical evaluation in epilepsy.
