# Correction: ICA Extracts Epileptic Sources from fMRI in EEG-Negative Patients: A Retrospective Validation Study

**DOI:** 10.1371/annotation/8eca54ef-1357-41d2-8026-8c939daba252

**Published:** 2014-01-07

**Authors:** Borbála Hunyadi, Simon Tousseyn, Bogdan Mijović, Patrick Dupont, Sabine Van Huffel, Wim Van Paesschen, Maarten De Vos

This article was republished on January 2, 2014, because of missing and incorrect equations. The publisher apologizes for the errors. Please download this article again to view the corrected version.

